# Postoperative accuracy quantification of corrective osteotomies: standardisation of Q3D-CT methodology

**DOI:** 10.1007/s00068-024-02684-8

**Published:** 2025-01-24

**Authors:** Sander J. C. Tabernée Heijtmeijer, Anne M. L. Meesters, Nico J. J. Verdonschot, Paul C. Jutte, Job N. Doornberg, Peter A. J. Pijpker, Joep Kraeima

**Affiliations:** 1 3D-Lab, University Medical Centre Groningen, University of Groningen, Groningen, The Netherlands; 2 Department of Oral and Maxillofacial Surgery, University Medical Centre Groningen, University of Groningen, Groningen, The Netherlands; 3 Department of Orthopaedics, University Medical Centre Groningen, University of Groningen, Groningen, The Netherlands; 4 Department of Trauma Surgery, University Medical Centre Groningen, University of Groningen, Groningen, The Netherlands; 5 Department of Orthopaedic and Trauma Surgery, Flinders Medical Centre, Flinders University, Adelaide, South Australia Australia; 6 Department of Biomechanical Engineering, University of Twente, Enschede, The Netherlands; 7 Orthopaedic Research Laboratory, Radboud Institute for Health Sciences, Radboud University Medical Center, Nijmegen, The Netherlands

**Keywords:** 3D technology, Accuracy, Osteotomy, Patient-specific, Validation, Virtual surgical planning

## Abstract

**Purpose:**

Currently, no gold standard exists for 3D analysis of virtually planned surgery accuracy postoperatively. The aim of this study was to present a new, validated and standardised methodology for 3D postoperative assessment of surgical accuracy in patients undergoing 3D virtually planned and guided corrective osteotomies.

**Methods:**

All patients who underwent 3D planned corrective osteotomy in 2021–2022 at our center with a postoperative CT were included. Postoperative surgical outcome was analysed with a postoperative CT and compared to the preoperative virtual surgical planning to determine achieved accuracy. Validation of the analysis was performed by evaluating the individual assessment of six experienced observers. A postoperative quantification was performed according to the proposed innovative methodology based on rotation axes of a virtual postoperative bone model aligned to the virtual preoperative bone model and virtual surgical planned bone model. To evaluate the intra-observer variability, one observer performed the assessment twice.

**Results:**

Quantification of 13 patients according resulted in measurements with a median range (and its interquartile range) for 3D translation of: 2.43 mm (3.17), for the angle deviations: 3D rotation, 2D coronal, 2D sagittal and 2D axial were: 0.66° (1.66°), 0.74° (0.44°), 0.99° (1.27°), 2.37° (5.00°), respectively. The inter- and intraobserver reliability established with the Intraclass correlation coefficient was for all measurements excellent (> 0.76).

**Conclusion:**

The proposed 3D CT technique provides an significant more accurate and objective method for assessment of surgical outcome of a guided corrective osteotomy. The present proposed novel methodology showed excellent inter- and intra-observer reliability with clinically acceptable absolute surgical outcome measurements.

**Supplementary Information:**

The online version contains supplementary material available at 10.1007/s00068-024-02684-8.

## Introduction

Bone deformations are a common cause for loss of function and pain. Because of a pathoanatomy patients may experience joint instability, and or increased risk of osteoarthritis [1]. The treatment often is a surgical correction of the bone, a corrective osteotomy. This procedure can be challenging to perform due to the common three-dimensional (3D) nature of the deformities [2, 3]. Traditionally, a corrective osteotomy was performed ‘free hand’ with assistance of two-dimensional (2D) preoperative x-ray images and intraoperative fluoroscopic imaging. In clinical practice, however, there is a trend toward 3D guided surgery, especially for multiplanar corrections. Recent advances in 3D technology and 3D printing introduce a promising adjunct: Surgery with the use of patient-specific instrumentation (PSI) to perform a guided corrective osteotomy based on a virtual surgical plan (VSP) [4–6]. PSI guided surgery leads to a superior clinical outcome for complex multi-planar corrections and the execution to correct bone deformities seems to be more predictable and accurate with these patient-specific 3D-printed guides compared to the ‘free hand’ method [7, 8]. 

Although there is an increasing number of studies published on corrective osteotomies performed with PSI, the number of articles that include a 3D postoperative surgical accuracy analysis remains limited. Usually, follow-up still consists of clinical examination and 2D radiographic assessment [9, 10]. A postoperative CT-scan is rarely performed, although evidence advocates that a 3D analysis is needed for an accurate and objective correlation with clinical outcome [11]. Corrective osteotomies of the proximal tibia, for example, require of traditional methods an accuracy < 3° for good long-term clinical outcomes [12]. Thus, to quantify surgical outcomes, the measurement error must be well below that threshold. A recent systematic review by Meesters et al. (2023)[8] showed that 6% of the 3D guided forearm osteotomies patients had complications after surgery. Almost 2% of the patients had to have a revision corrective osteotomy. A full 3D analysis may not be necessary for all patients, as 2D X-ray follow-up is often sufficient. However, in cases where issues arise, a 3D method becomes valuable for a comprehensive evaluation. The scarce number of articles that contain a 3D postoperative analysis of the surgical procedure use various methods, and do not assess their methods performance [13–18]. In oral- and maxillofacial surgery, for orthognathic surgery, corrective osteotomies of the upper or lower jaw there exists a validated 3D assessment tool, but it is specifically designed for that field and not suitable for corrective osteotomies in orthopaedics or trauma surgery [19]. In summary, despite the need, there is currently no gold standard for a postoperative 3D accuracy analysis. An accurate postoperative analysis could help to quantify the technical success of the surgery and distinguish postoperative residual symptoms from other potential causes, like soft tissue complications. Another benefit of an accurate postoperative analysis could be that the clinical recovery of a patient after surgery could be predicted more reliably in advance. In addition, large-scale randomised trials are required to prove that surgery in conjunction with a 3D VSP and PSI is superior compared to the conventional ‘free hand’ osteotomy. These trials will need an accurate surgical performance analysis [4]. Therefore, the establishment of a standardized 3D methodology for surgical outcome assessment is essential for accurate and reliable measurements.

The purpose of this study is twofold: first, to conduct a thorough assessment of the current methodology for quantification of the error between virtual surgical planning and obtained surgical result within our center to determine the relevance of the problem and review current 3D methodology. Second, to present and validate a novel methodology for assessment of the error between virtual surgical planning and obtained surgical result for 3D guided corrective osteotomies with clinically acceptable measurements and excellent inter- and intra-observer reliability.

## Methods

### Review current methodology within our centre

Five in-house observers, technical physicians with several years of experience with using virtual surgical planning (VSP) technology and PSI, were asked to independently perform a postoperative surgical accuracy analysis for one single patient who underwent a high tibial osteotomy (HTO), to assess the current methodology for analysis of the accuracy of the postoperative corrected tibia compared to the preoperative VSP. The observers were given no further information on what the analysis should consist of. The observers analysed the same case and were blinded to each other’s method and results. They had access to the preoperative 3D VSP and a postoperative 3D model. Their approaches and measurements were compared and their performance was assessed.

### Proposed novel methodology

In agreement with the observers, a technique was developed in-house based on a combination of previous reported approaches and the outcomes of the review of the current methodology in our center. The novel methodology is based on measurements based on a center (center of gravity) and rotation axes (inertial axes), this approach was rated best by observers. The full novel methodology is described below.

**Fig. 1:**
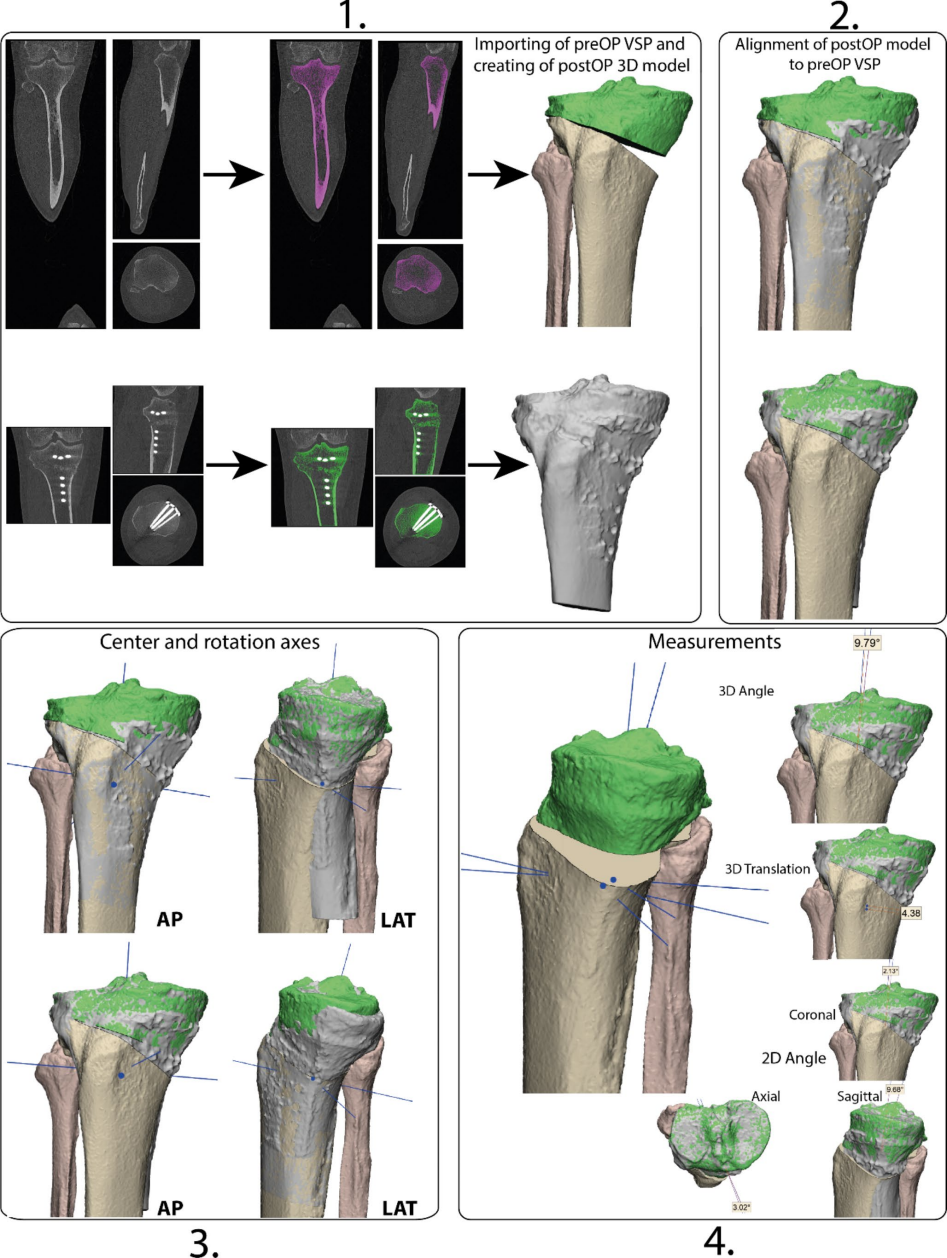
Workflow of the proposed novel methodology for assessing the postoperative accuracy of corrective osteotomies. Case 5 is taken as an example, which is a high tibial osteotomy (HTO). 1: First 3D models are created from a segmentation of a preoperative and postoperative CT-scan. The preoperative bone model is cut for an osteotomy and repositioned in a target position for surgery. The preoperative virtual surgical planning (top right) and a postoperative model (bottom right). 2: The 3D models are aligned in two ways, first to the preoperative state (bottom) and subsequently to the virtual planned corrected state (top). 3: An anterior-posterior (AP) and a lateral (LAT) view are visualised to clearly show the difference between both alignments. A center is determined and rotation axes are fitted to both postoperative models using the in-software automatic algorithm. 4: The difference between the axes and points can be seen. With the rotation axes, surgical accuracy angle measurements are be performed in a 3D coordinate system or in specific 2D anatomical coordinate systems. The centres are used to determine a 3D deviation measurement

The segmentation of the postoperative CT and creation of the 3D anatomical models was performed in Mimics (version 24.0, Materialise, Leuven, Belgium), 3D accuracy measurements were executed in 3-Matic (version 16.0, Materialise, Leuven, Belgium). The novel methodology consists of the following steps, which are also displayed in Fig. 1.

#### Step 1: creating the 3D postoperative model

The first step was to import the CT-scan. A scan was selected with a slice-thickness < 1 mm and with a bone kernel reconstruction. The bones were segmented with a global threshold based on the Hounsfield units of Bone (226–3071 HU). Region growing and manual editing tools were used if necessary to reduce noise and separate the bones from each other. When the segmentation was satisfactory, it was converted into a 3D model. The 3D model was then wrapped to get only the outer shell of the bones and remove internal structures.

#### Step 2: alignment of the preoperative VSP and postoperative model

Thereafter, the postoperative model was imported into the preoperative 3D VSP plan. The postoperative model was registered with the preoperative model of the bone, an iterative nearest point algorithm was used for the alignment [20]. The result of the ICP was visually confirmed. Bone surface not affected by the surgery were selected for the registration. The model will need to be aligned to the corrected and to the preoperative position of the treated bone.

#### Step 3: creation of center points and rotation axes

The next step was to create a centre point of rotation and three rotation axis per postoperative model, this could be done with an automatic algorithm in 3-Matic. A centre and rotation axes could be calculated based on the mesh of the models. Because the postoperative models are essentially duplicates that have been repositioned in space, the rotation axes are consistently generated in the exact same position relative to the bone models.

#### Step 4: measuring the surgical correction

The difference between the centres and the rotation axes of the models represents the surgical accuracy. 3D translation could be measured between the two points, 3D angle could be measured between the two rotational axes in the longitudinal direction. The 3D angle deviation could be split into 2D anatomical angle measurements to enhance clinical interpretation by measuring the angle between the projection of the lines on a predefined 2D anatomical plane. The longitudinal axes were used for the coronal and sagittal accuracy measurement and the transverse rotation axes were used for the axial rotation measurement. The planes created for preoperative planning could be reused for this purpose. These preoperative anatomical planes corresponded to weight-bearing radiographs used to determine the amount of correction or were made based on the International Society of Biomechanics recommendation for anatomical planes of joints [21, 22]. 

### Dataset

All patients aged 12 years and older, who were treated with 3D planned corrective osteotomies in 2021 or 2022 within our center were retrospectively included for this study, upon availability of a postoperative CT scan. CT scans were required to have a slice thickness < 1 mm and 50% overlap.

### Assessment of novel methodology

Six in-house observers independently performed a surgical accuracy analysis for all included patients according to the novel methodology. Observers were asked to determine the accuracy of the surgical outcome according to the standardized protocol and quantify it as 3D translation and rotation and additionally split the 3D angle into the 2D rotation components, in the three anatomical 2D planes. The postoperative CT scan was segmented by each observer individually, to account for variability between observers from this step onwards. All observers reported their measurements and they were again blinded to each other’s results. Their measurements were compared, and their performance was assessed. One observer performed the analysis again to determine intra-observer reliability, with a month between measurements to prevent confirmation bias. The average of the two measurements of this observer was used for the interobserver reliability analysis.

The primary outcomes of this study were postoperative accuracy measurements, inter- and intra-observer reliability, determined with the intraclass correlation (ICC) coefficient and differences from the median along with the range of the differences in quantification. The postoperative surgical accuracy was defined as: 3D angle deviation, and split into the coronal, sagittal and axial 2D angle component. Besides, 3D translation of the corrected bone.

### Statistical analysis

Statistical analysis was performed using IBM SPSS Statistics for Windows (version 28.0, Armonk, NY: IBM Corp). The accuracy data was presented as descriptive statistics, expressed as median and range for non-normal distributed data. Inter- and intra-observer reliability with a confidence interval of 95% was calculated with the ICC based on a two-way random model on absolute agreement. A *P*-value < 0.05 was considered to be statistically significant.

## Results

### Results of current methodologies

**Fig. 2:**
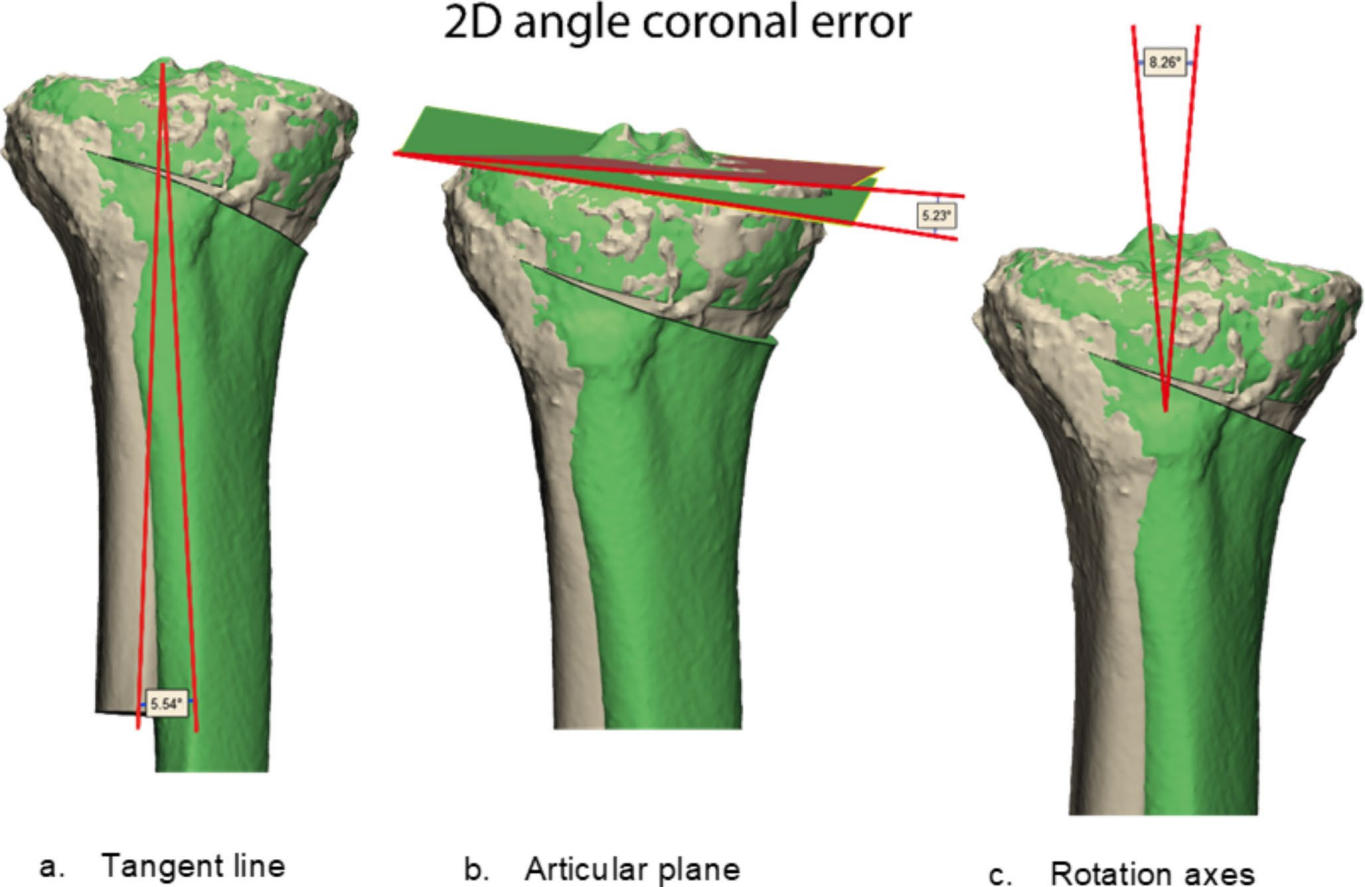
Representation of the three principal methods used for postoperative quantification of surgical performance. Planned surgical outcome in green, postoperative model in beige. This example shows the 2D coronal angle error A: Methodology based on using tangent lines. The observer placed two corresponding points and calculated the angle between both points and a point on the aligned part. B: Method based on the use of planes parallel to the articular surface. The observer fitted a plane to the postoperative realised articular surface and one to the planned surface, after aligning the postoperative model to the distal part. After creation of the planes the postoperative model was aligned to the proximal part with the plane of the model moving along. The deviation is now visible and can be measured. C: The last method based on rotation axes. The observer aligned the postoperative model to the preoperative model and created with the program automatically based on the surface mesh rotation axes. The deviation of the realised plan can be measured and visualized with corresponding rotation axes

Three different principal methodologies were used by the observers (*n* = 5) before introduction of the proposed novel protocol. The first method consisted of using tangent lines. The observer aligned the postoperative model to the proximal planned part of the tibia and after that set the 3D model in the correct anatomical view. The error from the preoperative planning was measured with 3D tangent lines, the angle between two corresponding points and a point on the proximal part, Fig. 2A. The second method was based on using planes, Fig. 2B. The observer aligned the postoperative model on the distal part of the shaft of the tibia. Then the articular surface was marked and an algorithm was used to create a parallel plane based on the marked surface. The observer did this for the postoperative tibia and for the planned tibial plateau. Then the postoperative model was aligned to the proximal part with the positioned plane moving along registration. The 3D deviation was measured by the angle between both planes and was split in 2D anatomical angles by measuring the angle between lines of the planes. The last method was based on rotation axes, Fig. 2C. The postoperative 3D model of the tibia was duplicated, and one model was registered to the proximal part of the planned tibia model and the other model was aligned to the distal part. Subsequently rotation axes were automatically created with the software based on the mesh of both postoperative models. The deviation was measured by the angle between similar rotation axis in 3D and on 2D anatomical planes.

#### Accuracy and inter- and intra-observer reliability of current methodology

**Fig. 3:**
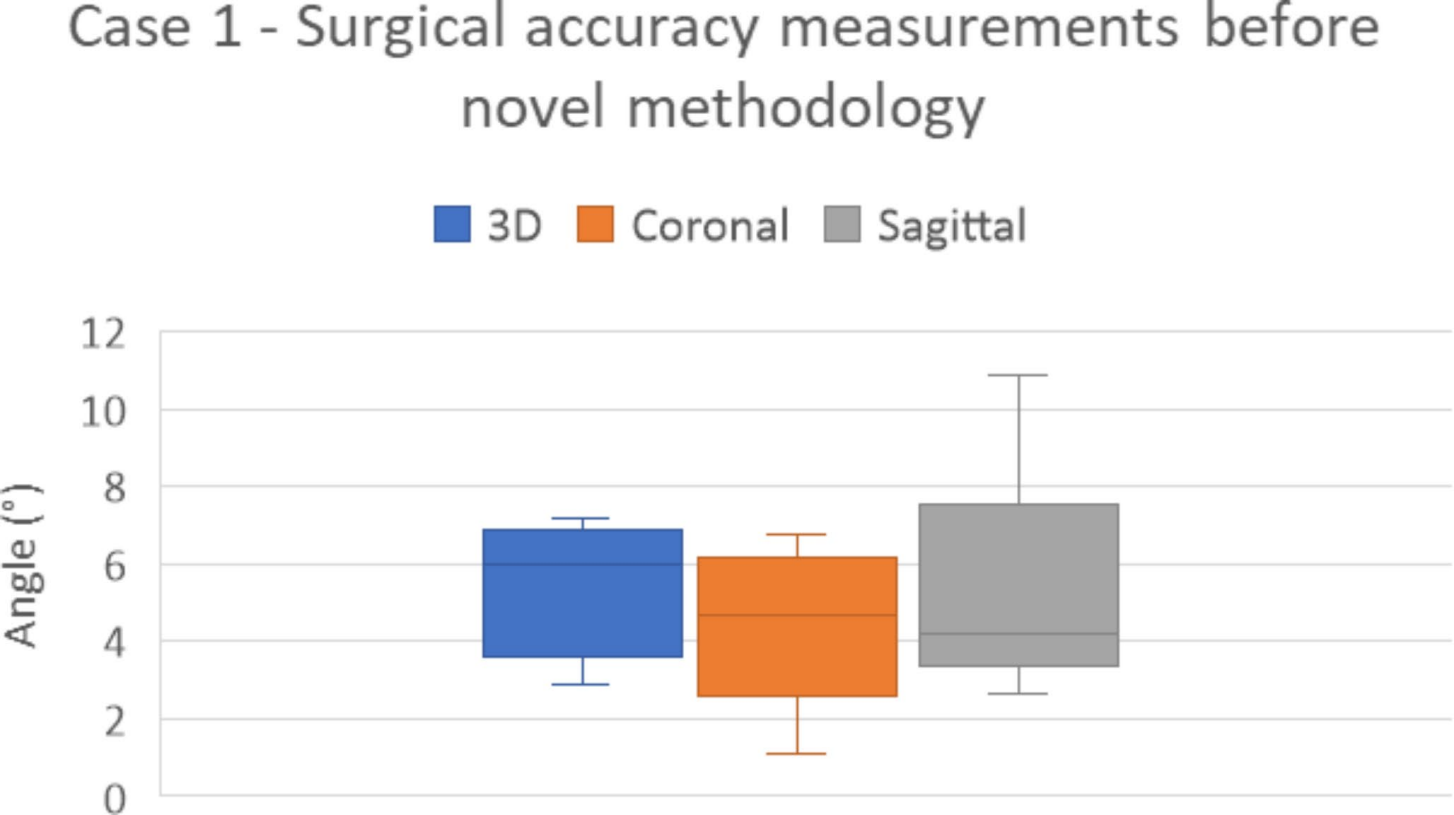
Results of the measurements of one HTO case (case 1) of the five observers before introduction of the novel methodology. Observers measured the 3D, 2D coronal and 2D sagittal angular deviation postoperatively from the planned position. The boxplot shows a range for all measurements, respectively, for 3D, 2D coronal and 2D sagittal: 2.88–7.15°, 1.09–6.75° and 2.67–10.84°

The measurements of the observers of the single case before implementing the novel methodology are shown in Fig. 3. All observers measured a 2D coronal and sagittal angular deviation, and except for one observer, all also measured the 3D deviation. However, as they used different methods, the angle was measured in a different coordinate system so it represents another angle and they cannot directly be compared. No one measured a translation. The range of the measurements was 4.27°, 5.66° and 8.17° for respectively 3D angular deviation, 2D coronal and 2D sagittal. The estimated ICC for all measurements was − 0.08 (95% CI: -0.13–0.95).

### Results of the novel methodology

**Fig. 4:**
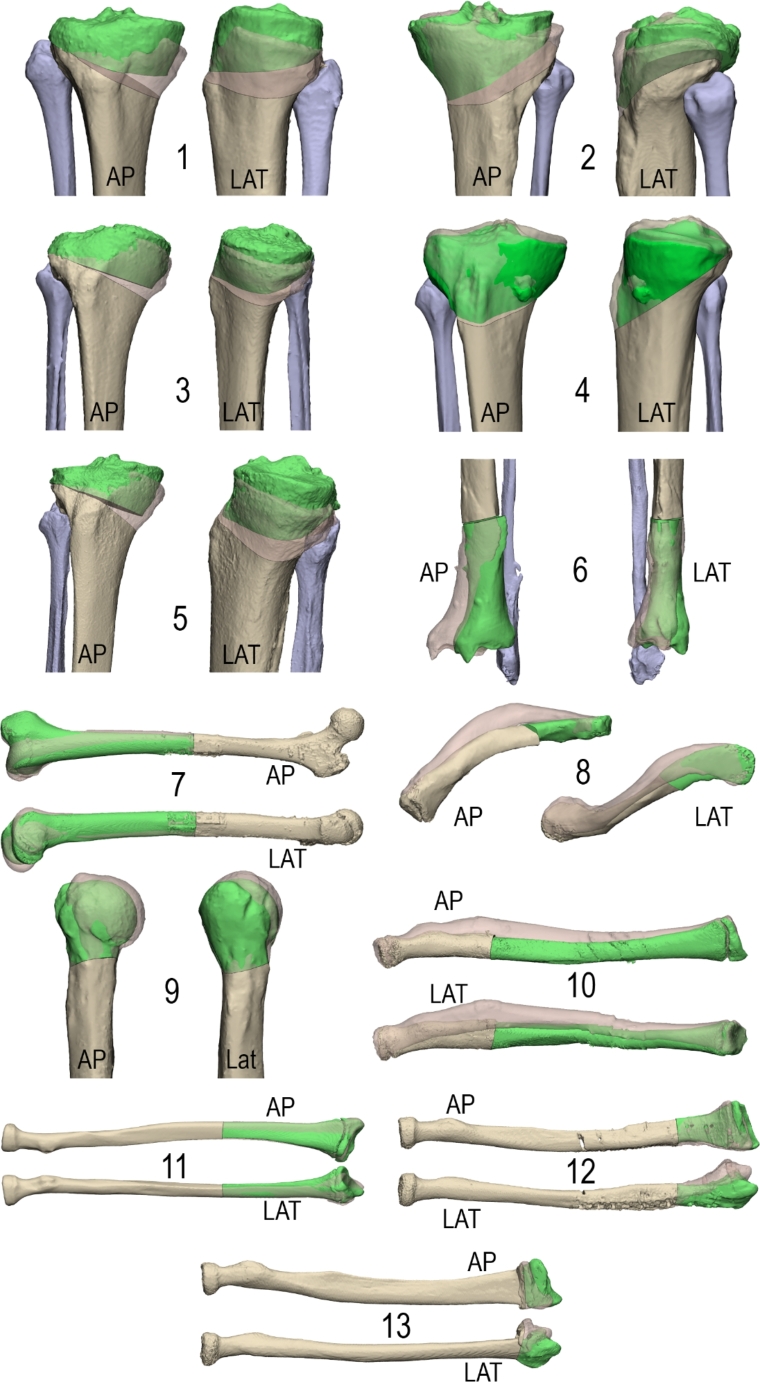
3D virtual surgical planning of all thirteen patients used for assessment. An *anterior*-*posterior* and lateral view of the treated bone is displayed. The virtually planned corrected position is shown in green and beige, the preoperative bone is transparent

Thirteen patients were retrospectively included for this study. Seven lower limb corrections (six tibia and one femur) and six upper limb corrections (four radius, one humerus and one clavicle) were included. Patient characteristics are shown in Table 1. All patients were treated with a 3D planned and guided corrective osteotomy and had a postoperative CT scan to assess surgical performance. Figure 4 shows the 3D virtual surgical planning of all cases. Case one was used for assessment of the current method.

#### Accuracy and inter- and intra-observer reliability of proposed novel methodology

The quantification measurements according to the novel methodology were accurate, the median range and its interquartile range was of 3D translation: 2.43 mm (3.17), for the angle deviations 3D rotation, 2D coronal, 2D sagittal and 2D axial: 0.66° (1.66), 0.74° (0.44), 0.99° (1.27), 2.37° (5.00) respectively. All results are shown in Table 2. The results for inter- and intra-observer differences showed excellent accuracy for postoperative evaluation of guided corrective osteotomies according to the novel methodology, all estimated ICC values are above 0.76.

**Table 1:** Patient characteristics of all included patients. Cases are split into lower limb and upper limb. The sex of the patient and the age at time of the surgery are shown. The area of the deformity is displayed and the type of deformity and the planned correction

	Case	Sex	Age at time of Surgery (y)	Area of deformity	Deformity	Planned correction
***Lower limb***	1	M	24	Proximal Tibia	Increased tibial slope	Closed wedge
	2	M	17	Proximal Tibia	Varus; Increased tibial slope	Open wedge
	3	F	27	Proximal Tibia	Increased tibial slope; Varus	Open wedge
	4	M	24	Proximal Tibia	Increased tibial slope	Closed wedge
	5	M	58	Proximal Tibia	Increased tibial slope; Varus	Open wedge
	6	F	64	Distal Tibia	Varus; Rotation	Closed wedge
	7	F	28	Mid-shaft Femur	Rotation	Rotation
***Upper limb***	8	M	16	Clavicula	Angulation; Shortening	Closed wedge
	9	M	23	Proximal Humerus	Varus; Rotation	Closed wedge
	10	M	12	Mid-shaft Radius	Angulation	Closed wedge
	11	F	16	Distal Radius	Rotation	Rotation
	12	M	17	Distal Radius	Volar angulation	Open wedge
	13	M	59	Distal Radius	Volar angulation	Open wedge

**Table 2:** Inter- and intra-observer variability of the novel methodology of all thirteen cases. The median range of the quantification of surgical accuracy measurements is shown along with interquartile range (IQR), the intraclass correlation (ICC) is shown with the 95% confidence interval (CI)

	Parameter	Measurement	Median range (IQR)	ICC (95% CI)
*Novel Methodology*	Inter-observer	3D translation	2.43 mm (3.17)	0.89 (0.79–0.96)
		3D rotation	0.66° (1.66)	0.85 (0.71–0.94)
		2D coronal	0.74° (0.44)	0.93 (0.86–0.98)
		2D sagittal	0.99° (1.27)	0.76 (0.58–0.90)
		2D axial	2.37° (5.00)	0.84 (0.70–0.94)
	Intra-observer	3D translation	0.32 mm (0.47)	0.97 (0.91–0.99)
		3D rotation	0.08° (0.50)	0.99 (0.96–1.00)
		2D coronal	0.16° (0.37)	0.95 (0.85–0.98)
		2D sagittal	0.14° (0.66)	0.98 (0.95–1.00)
		2D axial	0.50° (1.07)	0.93 (0.78–0.98)

## Discussion

This study aimed to evaluate current measurement methods and subsequently develop and evaluate a standardised method to perform a postoperative 3D accuracy analysis after a corrective osteotomy. The in-house assessment of the current methodology clearly demonstrated the need for a standardized validated methodology. The measurements were not clinically useful because of the wide range and there was no agreement among observers. The results of the assessment of the proposed novel methodology with measurements based on rotation axes, showed a significant improvement of the range and an excellent observer variability. The high estimated ICC demonstrates that the novel methodology is observer independent. Together, this makes the novel methodology an excellent validated technique for 3D surgical outcome assessment.

The internal assessment of the current methodology clearly demonstrated the relevance of the problem. Current measurements performed in our center without a protocol resulted in clinically unusable measurements. Observers used various techniques, which contributed to the wide range of measurements. Because of the differences in quantification of the surgical accuracy, the quantifications were not clinically usable, respectively, for 3D, 2D coronal and 2D sagittal: 2.88–7.15°, 1.09–6.75° and 2.67–10.84°. The ICC of -0.08 indicated no reliability among observers. For example, it was impossible with these ranges for the HTO case to assess surgical accuracy < 3°, to determine long-term postoperative outcomes. The current methodology resulted in unusable measurements for determining patient outcomes based on postoperative CT after a rehabilitation course and were therefore clinically irrelevant. Differences in quantification between observers will be even greater in clinical reality, as measurements were performed with one set of provided postoperative models. The added steps of segmentation and 3D modelling will result in additional variance, emphasizing the need for a uniform user independent 3D evaluation methodology even more. The inter-observer variability of measurements for a HTO case was significantly greater than the accuracy required for surgical correction as described in literature, despite the fact that all observers used methods outlined in the current literature [12]. Thus, the general consensus that when one takes the step from a 2D analysis to a 3D analysis, will lead to an improved result may not be the case. 3D analysis also requires a validated methodology to accurately quantify surgical accuracy.

The results of the proposed novel methodology showed a significant improvement. The median range of the measurements was of 3D translation: 2.43 mm (3.17), for the angle deviations 3D rotation, 2D coronal, 2D sagittal and 2D axial respectively: 0.66° (1.66), 0.74° (0.44), 0.99° (1.27), 2.37° (5.00). The inter- and intra-observer variability was excellent, high intraclass correlation coefficients (> 0.76) were determined for the standardised quantification approach.

However, the 2D measurements in the axial plane showed larger differences compared to the other measurements. The median range of 2.37° (IQR: 5.00°) is close to the traditional required accuracy of < 3° for determining long-term clinical outcome of HTO’s. This difference could be explained by the fact that long bones contain relatively few landmarks that can be used for alignment of the axis around which the axial rotation is quantified. The length of the long bones increases the accuracy of the alignment in the other planes. In addition, the postoperative CTs were not always of the entire bone, so part of the available information for registration was even not available, which resulted mainly for the axial rotation in inaccuracies. Furthermore, for the novel method, there is a clear difference between the inter-observer measurements and the intra-observer measurements. The difference between observers can only be explained by the 3D models created or by the alignment of the models, because the axes used to measure the deviation are automatically determined by the software based on the models. It is hypothesised that the difference between the intraobserver and interobserver measurements is explained by both, the 3D modelling and the alignment. Segmentation of case 8 was explained by the observers as challenging due to the metal artefacts which covered a large part of the clavicle, this case can also explain why the interobserver 2D sagittal measurement had a lower ICC than the other measurement. The artefacts resulted in a less accurate postoperative 3D model, making alignment specific in the sagittal plane for this case more challenging.

This is the first paper to the best of our knowledge to propose a standardised methodology for a 3D postoperative accuracy analysis after a corrective osteotomy, thus this study fills a knowledge gap. No research has been done before on the accuracy of a 3D postoperative analysis for guided corrective osteotomies but various methods have been described and used in the current literature. For example, measurements are performed with tangent lines positioned on 3D models [13, 14]. Others use specific landmarks to determine the accuracy [15]. A more advanced method consists of a transformation matrix between planned and achieved positioning, expressed in translation and rotation in a 3D anatomical coordinate system, although they use various reference centres [16–18]. Some use the centre point of the deviation, others use the center point of the 3D anatomical model part adjacent to the symptomatic joint. The proposed methodology was developed based on the results of an in-house assessment of the current methodology and in consultation with observers. Currently, there is no gold standard methodology for 3D quantification, which limits the comparison possibilities. Therefore, our proposed method was only compared with the existing method in our hospital, which served as a control group. The lack of a standardised method within our institution complicated direct comparisons, making it challenging to draw definitive conclusions on the relative effectiveness of the methods.

This study has some limitations. Despite the standardised method, observers might have to go through a learning curve, which was not investigated in this paper. The quantification measurements were conducted only by technical physicians, as they perform this type of measurement in our center. However, VSPs in other centres are performed by individuals with other backgrounds as well. It is expected that individuals from different backgrounds but also with experience and affinity with VSP technology, will also be able to perform these measurements according to the novel methodology, such as orthopaedic and trauma surgeons themselves or engineers. This could be evaluated in a follow-up investigation of the novel methodology, in which the effect of measurements of patients in other centres can be analysed as well. The study was conducted in a single centre, therefore the accuracy of measurements in other centres could be different, better or worse. As the study was conducted in one of the largest 3D labs of the academic medical centres in the Netherlands with more than 10 years of experience in virtual surgical planning, we expected that the problem of significant differences in accuracy assessment will only be greater in other smaller less experienced centres. We also expect that the raw data had an impact on the quality of the accuracy measurements. The quality of the CT scan and the software used will have been a factor in the outcomes and it will therefore need to be investigated whether the proposed methodology provides accurate measurements for other quality of scans as well. Case 8 already showed in this study that the quality of the bone segmentation impacted the accuracy of the measurements. The quality of a bone segmentation is inherently related to the quality of the CT scan.

It was hypothesised beforehand that inter- and intraobserver differences would be smaller when there are higher quality CT scans, scans of the entire postoperative bone, made with metal artefact reduction algorithms, though this could not yet be demonstrated with the current data. There are many variants of corrective osteotomies, but the performance of the novel methodology has only been evaluated for thirteen patients, although the patient group was diverse, only one or a few cases of a particular type of bone correction were included. Further studies with larger sample sizes should strengthen that this method results in consistent and accurate measurement and establish that for other osteotomies this method is clinically accurate as well. This article also did not study the clinical outcomes of the patients, in order to focus and keep the focus on the measurement method. But as a result, measurement accuracy cannot be correlated with clinical outcomes. While it is challenging to find any correlation with this, especially with this small group of patients, also the position of the bones does not determine the clinical outcome alone. Therefore in this article, the aim was not yet to identify clinical correlation. Though, Clinical outcomes have previously been published on a large proportion of the patients of this study by Assink et al. [23] The novel methodology may be less suitable or accurate for corrections of different bone types. Furthermore, the method should ideally also be implementable with publicly accessible software, as the analysis is currently performed with medically certified, commercially available software. Performing ICP alignment and determining rotation axes is often feasible with publicly accessible software. The current reliance on specialised software may limit the ability of other centres to perform the analysis. However, we expect that the use of commercially available, certified medical software helped reduce the time required for analysis and probably improved the quality of measurement. Follow-up studies are needed to investigate the exact impact of the software choice on the analysis results. Other requirements for a new gold standard is that the method should be simple and rapid to perform, however this was not measured or assessed by the observers during the measurements. The aim was first to find and validate a method for accuracy and observer reliability. In follow-up research, time required for the methodology and user-friendliness will also be included. In addition, efforts will be made to further automate the method to improve speed and ease of use even higher.

In the future, although not validated in this article, the novel methodology could also be used for postoperative assessments after surgery without the use of 3D planning or for preoperative analysis of malunions or reduced fractures. One can perform an assessment using the proposed standardized approach if a CT scan is available with adequate resolution for both the damaged and contralateral bone, or using a statistical shape model in case the contralateral side has not been scanned or is affected. The proposed and validated method also allows comparing results from different studies or between different centres. Namely, this method provides a single objective measurement of surgical outcome. Currently the methodology assess the postoperative alignment of the bone compared to the preoperative planned position. But the accuracy of the osteotomy plane compared to the preoperative VSP could also be a measurement of clinical relevance. Whether this is the case and how it should be measured could be further explored. The proposed methodology is in principle independent of the type of imaging. It only requires 3D models, which can also be made from other imaging modalities, e.g. bone MRI sequences or 3D reconstructions from 2-directional X-rays or ultrasound. In future research, the methodology can be validated for 3D models derived from other imaging modalities. Finally, it would be much more beneficial for patients to do an assessment intraoperatively already, postoperatively is actually too late should there still be a significant deviation. In the future, the method can be validated for intraoperative use, as it is in principle suitable for this purpose and can be applied when intraoperative imaging is available to create the necessary 3D models for the methodology.

In conclusion, various unvalidated methods are used for a 3D postoperative surgical outcome analysis of guided corrective osteotomies, the in-house assessment of the current methodology clearly showed the need for a standardised approach. The proposed novel methodology resulted in significant more accurate measurements with excellent inter- and intra-observer reliability. Therefore, the novel methodology is suitable and should be further validated outside our center as a 3D approach for postoperative analysis of corrective osteotomies.

## Electronic supplementary material

Below is the link to the electronic supplementary material.

Supplementary Material 1

## Data Availability

Raw measurement data is provided within the supplementary information files.
